# Astrocyte-Derived TGFβ1 Facilitates Blood–Brain Barrier Function via Non-Canonical Hedgehog Signaling in Brain Microvascular Endothelial Cells

**DOI:** 10.3390/brainsci11010077

**Published:** 2021-01-08

**Authors:** Jiyang Fu, Liang Li, Dong Huo, Shuli Zhi, Ruicheng Yang, Bo Yang, Bojie Xu, Tao Zhang, Menghong Dai, Chen Tan, Huanchun Chen, Xiangru Wang

**Affiliations:** 1 State Key Laboratory of Agricultural Microbiology, College of Veterinary Medicine, Huazhong Agricultural University, Wuhan 430070, China; fujy@webmail.hzau.edu.cn (J.F.); liangli@webmail.hzau.edu.cn (L.L.); hdhznydx@webmail.hzau.edu.cn (D.H.); shellyzhi@webmail.hzau.edu.cn (S.Z.); yangruicheng@mail.hzau.edu.cn (R.Y.); ybtc@webmail.hzau.edu.cn (B.Y.); pochieh@webmail.hzau.edu.cn (B.X.); taozhang18@mails.jlu.edu.cn (T.Z.); daimenghong@mail.hzau.edu.cn (M.D.); tanchen@mail.hzau.edu.cn (C.T.); chenhch@mail.hzau.edu.cn (H.C.); 2 Key Laboratory of Preventive Veterinary Medicine in Hubei Province, The Cooperative Innovation Center for Sustainable Pig Production, Wuhan 430070, China; 3 Key Laboratory of Development of Veterinary Diagnostic Products, Ministry of Agriculture of the People’s Republic of China, Wuhan 430070, China; 4 International Research Center for Animal Disease, Ministry of Science and Technology of the People’s Republic of China, Wuhan 430070, China

**Keywords:** blood–brain barrier, TGFβ1, hedgehog signaling, Gli2, ZO-1

## Abstract

The blood–brain barrier is a specialized structure in mammals, separating the brain from the bloodstream and maintaining the homeostasis of the central nervous system. The barrier is composed of various types of cells, and the communication between these cells is critical to blood–brain barrier (BBB) function. Here, we demonstrate the astrocyte-derived TGFβ1-mediated intercellular communication between astrocytes and brain microvascular endothelial cells (BMECs). By using an in vitro co-culture model, we observed that the astrocyte-derived TGFβ1 enhanced the tight junction protein ZO-1 expression in BMECs and the endothelial barrier function via a non-canonical hedgehog signaling. Gli2, the core transcriptional factor of the hedgehog pathway, was demonstrated to modulate ZO-1 expression directly. By the dual-luciferase reporter system and chromatin immunoprecipitation, we further identified the exact sites on Smad2/3 that bound to the *gli2* promotor and on Gli2 that bound to the *zo-1* promotor. Our work highlighted the TGFβ1-mediated intercellular communication of astrocytes with BMECs in BBB, which shall extend current knowledge on the BBB homeostasis physiologically, and more importantly suggests TGFβ1 as a potential effector for future prevention and amelioration of BBB dysfunction.

## 1. Introduction

The blood–brain barrier (BBB), a specialized structure in mammals, separates the brain from the bloodstream and maintains the homeostasis of the central nervous system (CNS) by regulating the influx and efflux of ions, oxygen, and nutrients between the blood and brain compartments and restricting invasion of the toxins and pathogens [1,2,3]. This barrier is mainly composed of brain microvascular endothelial cells (BMECs), astrocytes, and pericytes. Among these, BMECs act as the direct barrier units, which are characterized by the presence of tight junction (TJ) proteins (such as ZO-1, occludin, and claudin-5) and high trans-endothelial electrical resistance (TEER) [4,5,6]. The reduction of TJ protein expression will result in BBB integrity disruption, which is an important indicator of CNS dysfunction [7,8,9]. Meanwhile, astrocytes are recognized as the essential supporter of the BMECs. It has been demonstrated that many factors from astrocytes participate in modulating endothelial properties in BBB, and thus maintaining the barrier environment. For instance, astrocyte-derived GDNF promoted the TJ formation in BBB and supported the survival of neurons [10]. The Kir4.1 and AQP4, located in the end-feet of astrocytes, interacted with endothelial cells to regulate water influx and efflux of brain [11]. Astrocyte-derived angiotensin I and angiotensin II acted on the type 1 angiotensin receptors in endothelial cells to keep low infiltration of BBB [12]. These reports suggest that the normal communication between astrocytes and BMECs is critical for the BBB structural and functional integrity.

Transforming growth factor β1 (TGFβ1), one of the transforming growth factor β (TGFβ) family members, is a pleiotropic cytokine and plays important roles in multiple pathological and physiological processes [13]. Generally, TGFβ1 binds to TGFβ receptor type II (TGFBRII) and TGFβ receptor type I (TGFBRI) to initiate the signaling, and the downstream transcription factor Smads translocates to the nucleus to modulate target genes’ transcription [14,15,16,17]. Such TGFBRs/Smads signaling has been reported to participate in neuronal migration, neuron survival, and astrocyte differentiation in CNS [18,19]. In other biological process, the TGFβ1 pathway was also reported to function through cross-talking with other signaling pathways. For instance, TGFβ1 activated the Notch signaling in hepatic stellate cells by increasing the expression of *notch1*, *jagged1*, and *hes1*, thereby regulating the liver microenvironment [20]. Additionally, in chondrocytes, TGFβ1 promoted connexin43 expression through activating the ERK-MAPK pathway, which mediated gap junction intercellular communication in chondrocytes, especially in high cell intensity [21]. The cross-talking between TGFβ1 signaling and other pathways has also been identified in multiple processes, such as cancer invasion, stem-cell self-renewal, and lineage-specific differentiation [22]. Whether TGFβ1 cross-talks with other signaling involved in the BBB is still not well known and worth pursuing.

Hedgehog signaling is conserved among multiple species and plays important roles in many aspects of physiological and pathological processes. The canonical hedgehog signaling is activated by three secretory ligands, Shh, Ihh, and Dhh, and the intercellular signaling is transduced by transcription factors Gli1, Gli2, and Gli3 [23]. In CNS, the hedgehog pathway determines the formation and development of the neural tubes. High concentration of Shh in brain was reported to activate *nkx6.1*, *olig2*, and *nkx2.2* genes, thereby promoting the development of the nervous system [24]. For adult animals, the hedgehog signaling was also required for neural stem cells so as to maintain their differentiation ability to replenish new neurons [25]. Besides, hedgehog signaling was also reported to maintain BBB immune quiescence through modulating Wnt signaling in BMECs [26]. Notably, the hedgehog signaling cross-talking with TGFβ1 cascades has been reported in cancer development and metastasis [27]. We wondered whether this cross-talking between hedgehog signaling and TGFβ1 cascades influences the BBB’s function.

In this study, we report the astrocyte-derived, TGFβ1-mediated intercellular communication of astrocytes with BMECs via non-canonically activated hedgehog signaling. The TGFβ1-triggered Smad2/3 activation in BMECs increased the expression of Gli2, the key transcription factor of hedgehog signaling. Gli2 bound to the zo-1 promotor and enhanced the ZO-1 expression, and thus contributed to the BBB’s maintenance. Herein, we shed light on the TGFβ1-mediated intercellular cross-talking between astrocytes and brain endothelium. This finding shall broaden the existing knowledge regarding the homeostasis of the BBB, and may also help to further improve the treatment strategies for the BBB dysfunction.

## 2. Materials and Methods

### 2.1. Cell Culture

The human brain microvascular endothelial cells (hBMECs) were kindly gifted from Prof. Kwang Sik Kim in Johns Hopkins University School of Medicine, and routinely cultured in RPMI 1640 supplemented with 10% fetal bovine serum (FBS), 2 mM L-glutamine, 1 mM sodium pyruvate, essential amino acids, nonessential amino acids, vitamins, and penicillin and streptomycin (100 U/mL). The astrocyte cell line U251 (a kind gift from Prof. Shengbo Cao in Huazhong Agricultural University, Wuhan, China) and HEK-293T cells (ATCC^®^ CRL-3216™) were cultured in Dulbecco’s modified eagle’s medium (DMEM) with 10% FBS and penicillin and streptomycin (100 U/mL). All cells were cultured in 37 °C incubator under 5% CO_2_ until reaching monolayer confluence. In some experiments, confluent hBMECs were starved in serum-free medium (1:1 mixture of Ham’s F-12 and 199 medium) for 12–16 h before further treatment.

### 2.2. Reagents and Antibodies 

The TGFβ/Smads signaling inhibitors SD208 and LY2109761, and the hedgehog Gli1/2 inhibitor GANT61 were purchased from MedchemExpress (Princeton, NJ, USA). The immunofluorescence staining kits containing Cy3-labeled goat anti-rabbit IgG, and the 4′,6-diamidino-2-phenylindole (DAPI) reagent were obtained from Beyotime (Shanghai, China). Anti-Smad2, anti-Smad3, anti-phospho-Smad2 and anti-phospho-Smad3 antibodies were obtained from ABclonal Biotechnology Co., Ltd. (Boston, MA, USA). Anti-Gli1, anti-Gli2, anti-TGFBRI, anti-TGFBRII, anti-ZO-1, and anti-OCLN antibodies were from Proteintech (Chicago, IL, USA). The antibodies applicated in ChIP assays (Anti-Smad2/3, anti-Gli2), the HRP-conjugated anti-rabbit IgG antibody, HRP-conjugated anti-mouse IgG antibody, and SimpleChIP^®^ Plus Enzymatic Chromatin IP Kit (Magnetic Beads) were purchased from Cell Signaling Technology (Danvers, MA, USA). Anti-β-actin antibody was obtained from HuaAn Biotechnology Co., Ltd. (Hangzhou, China). The lipofectamine 3000 transfection reagent was obtained from Invitrogen (Carlsbad, CA, USA). Puromycin and transwell chambers were purchased from Corning (Corning, NY, USA). Gli1 and Gli2 CRISPR/Cas9 plasmids were synthesized from Nanjing YSY Biotech Co. LTD. (Nanjing, China). Human recombinant TGFβ1 and mouse recombinant TGFβ1 were obtained from R&D system (Minneapolis, MN, USA). Human TGFβ1 ELISA Kit was obtained from 4A Biotech (Beijing, China).

### 2.3. Mouse Assays

The 21-day-old specific-pathogen-free (SPF) female Kunming mice were obtained from the experimental animal center at China Three Gorges University (Hubei Province, China). Mice were injected with the recombinant TGFβ1 protein or SD208 through the tail vein at indicated dosages. The brains from moribund and control mice were subjected to Western blot assays.

The current study was carried out in accordance with the guidelines established by the China Regulations for the Administration of Affairs Concerning Experimental Animals (1988) and Regulations for the Administration of Affairs Concerning Experimental Animals in Hubei Province (2005). All procedures and handling techniques were approved by The Scientific Ethic Committee of Huazhong Agricultural University (Animal Welfare Assurance No. HZAUMO-2019-021).

### 2.4. hBMECs and U251 Co-Cultivation

The in vitro BBB co-culture model was constructed in the transwell chamber following methods previously reported [28]. In brief, hBMECs were pre-seeded in the up chamber of transwell inserts (6.5 mm diameter inserts, 3.0 μm pore size) at 8 × 10^3^ cells per well, and U251 astrocytes were synchronously seeded in the 24-wells plate. After 12 h of growth, the transwell inserts with hBMECs were transferred to the 24-wells plate containing U251, and the co-culture medium was changed into the hBMECs complete medium. During co-cultivation, the TEER values were detected with Millicell-ERS (MERS00002) instrument (Millipore, Burlington, MA, USA). 

### 2.5. Western Blot

Mouse brains or hBMECs cultures were homogenized or lysed in RIPA buffer containing protease inhibitor cocktail, and centrifuged at 15,000× *g* for 30 min at 4 °C to remove the insoluble cell debris. Protein concentrations of brain lysates or cell lysates were measured with BCA protein assay kit (NCM Biotech, Suzhou, China), and equivalent protein samples were subjected to Western blot assay as we previously described [29]. 

### 2.6. RT-PCR and qPCR

Total RNA was extracted by the TRIzol reagent (Thermo Fisher Scientific, Waltham, MA, USA), and the RNA purity and concentration were assessed by NanoDrop 2000 Ultramicro spectrophotometer (Thermo Fisher Scientific). RT-PCR was performed to generate cDNA by using HiScript II Q RT SuperMix for qPCR (+gDNA wiper) (Vazyme, Nanjing, China). The qPCR was performed with qTOWER^3^/G quantitative real-time PCR thermal cycler (Analytikjena, Jena, Germany) by using MonAmp SYBR Green qPCR Mix (Monad Biotech Co., Ltd., Wuhan, China) following the manufacturer’s instructions. The primers used for qPCR were listed in Table S1. Expression of the target genes was normalized against *gapdh*. Each assay was performed in triplicate.

### 2.7. Enzyme-Linked Immunosorbent Assay (ELISA)

U251 cells were mono-cultured or co-cultured with hBMECs, and the concentration of TGFβ1 in the culture supernatant was determined with a TGFβ1 ELISA Kit, following the manufacturer’s instructions. Briefly, the culture supernatant was collected and centrifuged at 12,000× *g* for 5 min at 4 °C. Before test, the samples were treated with 1 N HCl and 1.2 N NaOH/0.5 M HEPES to activate latent TGFβ1 to the immunoreactive form. The activated samples were added into 96-well plates and incubated at 37 °C for 2 h. Then, the biotin labeled detection antibody, streptavidin-HRP, TMB substrate solution, and stop solution were sequentially added into the plates. The optical density of each well was determined with a microplate reader set to 450 nm, and the concentration of TGFβ1 in the culture supernatant was obtained through the standard curve.

### 2.8. Immunofluorescence (IF)

For IF, cells were seeded and grown in glass-bottomed dishes (diameter 35 mm) for the specified treatment. Paraffin sections of the challenged mouse brains were deparaffinized and rehydrated in xylene and ethanol. IF experiments were performed according to the instructions provided by the relevant kits. Briefly, cells or sections were washed with PBS three times and then fixed with 4% paraformaldehyde for 30 min. The fixed cells or sections were then treated with 1% Triton X-100 in PBS prior to non-specific site blocking and antibody incubation. Here, Gli1, Gli2, ZO-1, and TGFBRI/II were labeled with Cy3, and CD31 was labeled with FITC. The cells in the dishes were finally mounted and visualized with Zeiss LSM 880 microscope (Carl Zeiss Jena, Jena, Germany), and sections were observed with ECHO REVOLVE microscope (Echo Laboratories, San Diego, CA, USA).

### 2.9. Electric Cell-Substrate Impedance Sensing

The electric cell-substrate impedance sensing (ECIS) Zθ system (Applied BioPhysics, Troy, NY, USA) was employed to monitor the barrier function of hBMECs with specific treatments as previously reported [30]. Briefly, cells were seeded on the collagen-coated and gold-plated electrodes in 96-well chamber slides (96W1E+) at 7 × 10^4^ cells per well and cultured until reaching confluence. The TEER was continuously monitored to reflect the formation of the barrier. After stable maximal resistance was reached, the specific reagents or treatments were added into the wells at indicated concentration, and the TEER changes were automatically monitored by the ECIS system. All data recorded in ECIS system were analyzed and normalized as the Rb values (Norm. Parameter Values), which representing the barrier function alteration along with time. Each treatment was performed with 5 parallel duplications.

### 2.10. CRISPR/Cas9 Genomic Editing 

For CRISPR/Cas9 editing in hBMECs, they were transfected with commercially synthesized CRISPR/Cas9 plasmids containing puromycin resistance gene. After 24 h of transfection, the medium was replaced and the positive-transfected cells were selected in the fresh medium containing puromycin for another 24 h. Surviving cells were transferred into 96-well plates with limiting dilution and incubated until single-cell clone was formed. Genomic DNA from each clone was extracted and subjected to PCR amplification, and PCR-positive editing cells were identified by sequencing.

### 2.11. Transfection

HEK-293T or hBMECs cells grown to 70% confluence were subjected to transfection experiments with Lipofectamine 3000 reagent according to the manufacturer’s instructions (Invitrogen, Waltham, MA, USA). Briefly, 5 μg of plasmids, 10 μL of P3000, 7.5 μL of Lipo3000, and 500 μL of Opti-MEM were mixed gently and incubated at room temperature for 15 min. The mixture was then added dropwise to the cells in the 6-well plates and incubated at 37 °C with 5% CO_2_ for 24 h. 

### 2.12. Dual-Luciferase Reporter Assay

Prior to luciferase reporter assay, the coding sequences (CDS) of human Smad2, Smad3, Gli1, and Gli2 were amplified and cloned into pcDNA3.1(+) vector to generate the overexpression plasmids pcDNA3.1-Smad2, pcDNA3.1-Smad3, pcDNA3.1-Gli1, and pcDNA3.1-Gli2. The promotor regions of *gli2* and *zo-1* were amplified and cloned into the firefly luciferase reporter vector pGL3-basic to generate the wildtype reporter plasmids pGL3-*gli2*-promo-WT and pGL3-*zo-1*-promo-WT. Meanwhile, a serial of truncated promoter and site-directed mutation of promotor were similarly constructed into pGL3-basic (Figures 4 and 5). All primers used in the dual-luciferase assays were listed in Table S2. 

For dual-luciferase reporter assay, the pcDNA3.1(+) overexpression plasmid, the corresponding pGL3 reporter plasmid, and the pRL-TK plasmid were co-transfected into HEK-293T cells in 24-wells plates. Both firefly luciferase activity and renilla luciferase activity were tested after 36 h of transfection by Dual-Luciferase Reporter assay system (Promega, WI, USA) with Spark 10M multimode microplate reader (Tecan, Männedorf, Switzerland). Relative luciferase activity was calculated by the ratio of reporter activity (firefly fluorescence) to that of control activity (renilla fluorescence), and the results were shown as the representative of three independent assays.

### 2.13. Chromatin Immunoprecipitation (ChIP)

ChIP was performed to test the interaction between transcription factors and its potential target genes by using SimpleChIP^®^ Plus Enzymatic Chromatin IP Kit (CST) following the manufacturer’s instructions. Briefly, cells in the dishes were fixed in formaldehyde to cross-link proteins with DNAs. Cells were next digested by micrococcal nuclease and subjected to the immunoprecipitation procedure. The products were treated with protease K and then subjected to DNA isolation. Purified DNA was used in the following qPCR/PCR amplification. The primers used for ChIP-qPCR/PCR were listed in Table S3.

### 2.14. Statistical Analysis

Data are expressed as the mean ± standard error of the mean (mean ± SEM) from at least three replicates. Statistical significance of the differences between each group was analyzed by a one-way analysis of variance (ANOVA) or two-way ANOVA embedded in GraphPad Prism, version 6.0 (GraphPad Software Inc., La Jolla, CA, USA). *p* < 0.05 (*) was considered significant, and *p* < 0.01 (**) was considered extremely significant. 

## 3. Results

### 3.1. Astrocyte-Derived TGFβ1 Enhanced the Barrier Function of BMECs 

**Figure 1:**
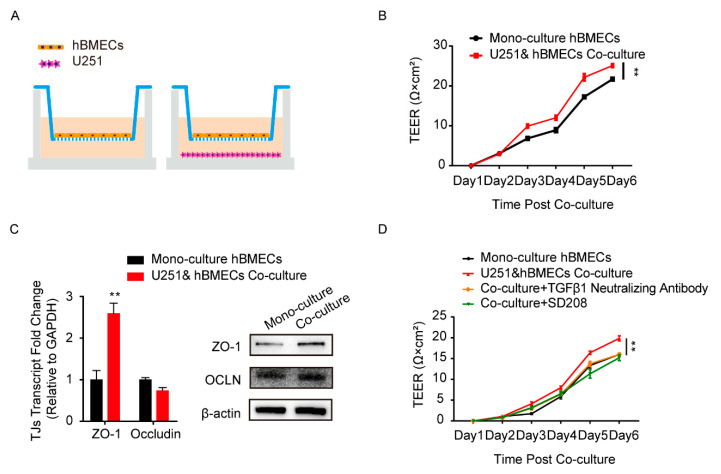
Astrocyte-derived TGFβ1 enhanced brain microvascular endothelial cells (BMECs) barrier function. (**A**) The human brain microvascular endothelial cells (hBMECs) mono-culture and co-culture model with U251. (**B**) Comparison of trans-endothelial electrical resistance (TEER) values of the hBMECs under both culture conditions. ** *p* < 0.01. (**C**) The expression of TJ proteins ZO-1 and occludin in hBMECs under both culture conditions via qPCR and Western blot. ** *p* < 0.01. (**D**) Effects of TGFβ1 blocking (with TGFβ1 neutralizing antibody at 10 μg/mL and with inhibitor SD208 at 10 μM) on the TEER values of hBMECs with co-culture. ** *p* < 0.01.

Astrocytes and BMECs are the major components of the BBB, and the communication between these two cell types is essential to the barrier function [31]. To simulate this cell-to-cell commutation in vitro, we constructed the co-culture model by seeding human BMECs (hBMECs) with/without U251 in the transwell chamber, as shown in Figure 1A. The TEER values of hBMECs under both culture conditions were monitored and compared, and results showed that the TEER values of the co-cultured hBMECs were significantly higher than that of the mono-cultured cells (Figure 1B), indicating a better barrier function of the monolayer endothelial cells. The TJ proteins in hBMECs under both conditions were also detected, and ZO-1 exhibited a significantly higher expression in co-cultured hBMECs (Figure 1C). These suggested that there might be certain effectors secreted from U251 that enhanced the ZO-1 expression in hBMECs, and promoted the endothelial barrier function. Previously, several proteins have been reported to be secreted by astrocytes [32,33], and together with our previous transcriptomic data and the secretory proteomic results, we suspected that TGFβ1 was one such astrocyte-derived effector modulating endothelial barrier function. We therefore tested the TGFβ1 generation in the culture supernatant of U251 cells, either with or without hBMECs co-cultivation. As presented in Figure S1A, the concentration of TGFβ1 tested by ELISA increased from less than 1 ng/mL at day 1 to around 10 ng/mL at day 6 (Figure S1A). The transcription and protein concentration of TGFβ1 in U251 were not differentiated between monolayer U251 and the co-culture conditions (Figure S1A,B). All that evidence together suggested that the astrocyte-derived TGFβ1 might play a role in the communication between U251 and hBMECs. To prove this hypothesis, we tried to block TGFβ1 in the transwell co-culture system with TGFβ1 neutralizing antibody and its downstream signaling inhibitor SD208. As shown, the higher TEER values of the co-cultured hBMECs significantly decreased to that of the mono-cultured cells by both antibody neutralization and inhibitor treatment (Figure 1D), which largely supported the participation of TGFβ1 in barrier enhancement.

### 3.2. TGFβ1 Promoted TJ Protein ZO-1 Expression via Smad2/3

**Figure 2:**
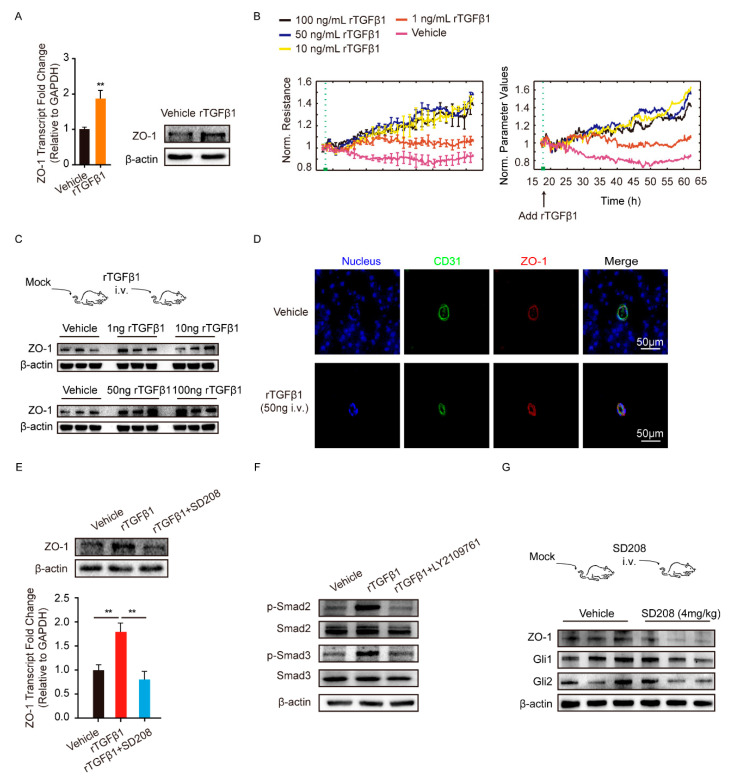
TGFβ1 treatment enhanced BMECs tight junction protein ZO-1 expression. (**A**) The rTGFβ1 treatment (50 ng/mL) on the expression of ZO-1 in monolayer hBMECs. ** *p* < 0.01. (**B**) The effect of rTGFβ1 treatment on hBMECs barrier resistance monitored via ECIS system. The assay was performed with 5 replicates. (**C**) The effect of rTGFβ1 injection on the ZO-1 expression in mouse brains (*n* = 3). The β-actin was used as the loading control for the blotting. (**D**) IF demonstrated the ZO-1 expression on BMECs of the mice in response to rTGFβ1 treatment (50 ng, i.v.). BMECs were marked with CD31 in green. Scale bars indicated 50 μm. (**E**) The effects of rTGFβ1 treatment (50 ng/mL) and TGFβ1 signaling on the expression of ZO-1 in hBMECs. The qPCR assays were performed in triplicate, and data are represented as mean ± SEM. ** *p* < 0.01. (**F**) The phosphorylation activation of Smad2 and Smad3 in hBMECs by the treatment of rTGFβ1 (50 ng/mL). (**G**) The effects of SD208 injection (4 mg/kg i.v.) on the expression of ZO-1, Gli1 and Gli2 in mouse brains (*n* = 3). The β-actin was tested as the loading control. Results of 3 mice in each group were randomly presented.

The tight junction protein ZO-1 played key roles in maintaining the endothelial barrier function. Herein, we employed recombinant TGFβ1 (rTGFβ1) at 50 ng/mL to stimulate monolayer hBMECs and observed that ZO-1 expression was significantly increased, compared with the vehicle treatment (Figure 2A). rTGFβ1 dose-dependently increased the barrier function of monolayer hBMECs tested by the electric cell-substrate impedance sensing (ECIS) system (Figure 2B). We also treated the mice with multiple dosages of rTGFβ1 by intravenous injection and found that TGFβ1 injection increased the ZO-1 expression in brains dose-dependently (Figure 2C and Figure S2A). We also observed that rTGFβ1 treatment upregulated ZO-1 in mice BMECs labeled with CD31 via IF (Figure 2D).

**Figure 3:**
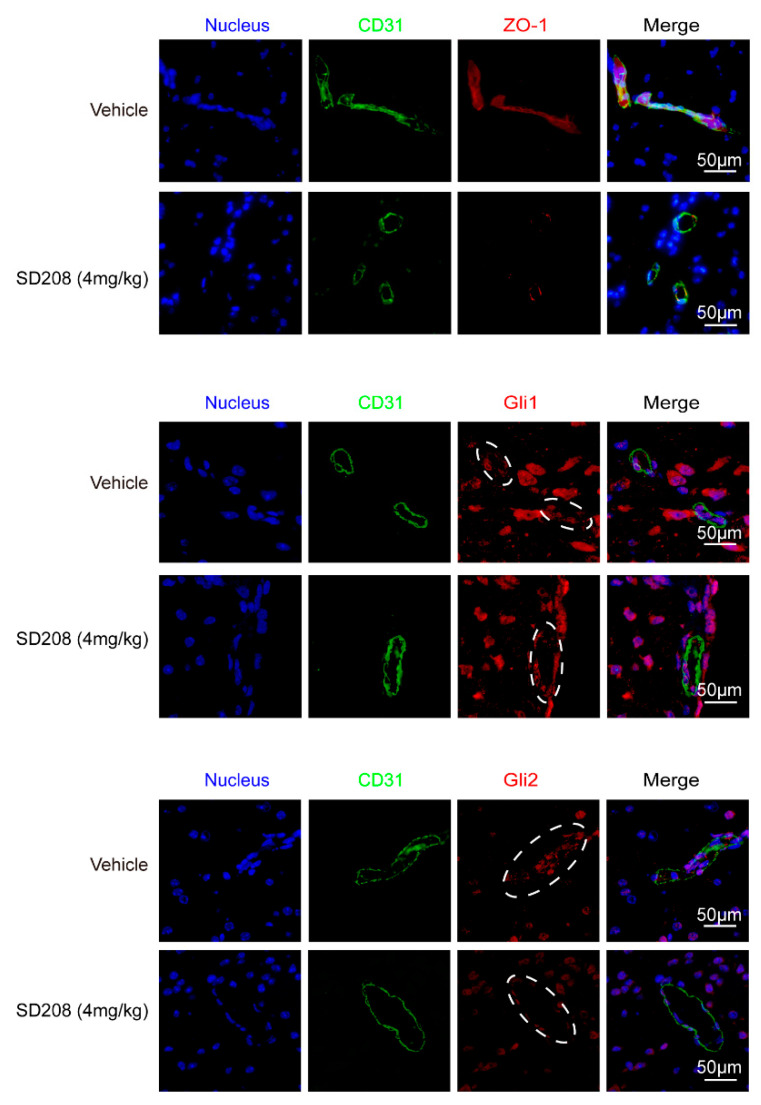
SD208 treatment decreased ZO-1 and Gli2 expression in mice BMECs. Mice were treated with SD208 at 4 mg/kg, and the expression of ZO-1, Gli1, and Gli2 was detected by IF. BMECs are marked in green (with CD31). Scale bars indicate 50 μm. The rings of doted lines indicate Gli1 or Gli2 expression in BMECs.

Next, we sought to investigate how TGFβ1 upregulated ZO-1 expression in hBMECs. Generally, TGFβ1 exerts its regulative roles via acting on its receptor TGFBRI/II and the subsequent activation of Smad2/3. As shown in Figure S1C, the TGFBRI/II were expressed at similar levels in hBMECs under both mono-culture and co-culture conditions. Additionally, we observed that the astrocyte-promoted TEER increase in hBMECs was significantly suppressed by TGFβ1 signaling axis inhibitor SD208 (Figure 1D), and rTGFβ1-induced ZO-1 upregulation in hBMECs was also suppressed by SD208 treatment at 10 μM (Figure 2E). Meanwhile, Smad2/3 phosphorylation in response to rTGFβ1 in hBMECs was also prevented by the TGFBRI/II inhibitor LY2109761 at 10 μM (Figure 2F and Figure S2B). These in vitro data indicated that the TGFBRI/II-Smad2/3 axis was involved in rTGFβ1 regulation of ZO-1. In vivo, the TGFBRI/II expression were also detected in BMECs labeled with CD31 (Figure S1D), and a 24-h treatment of SD208 at 4 mg/kg significantly attenuated the expression of ZO-1 in mouse brains (Figure 2G and Figure S2C). By IF, we also observed the decrease of ZO-1 in CD31 labeled BMECs upon SD208 treatment (Figure 3). These data further supported the concept that ZO-1 expression in hBMECs was regulated by TGFβ1-TGFBRI/II-Smad2/3 axis.

### 3.3. TGFβ1 Activated Non-Canonical Hedgehog Signaling to Enhance ZO-1 Expression

As is known, Smad2/3 functions via Smad2/3/4 complex and binds to the target gene promotor at a specific binding motif [13], as shown as Figure S3A. To quickly determine the Smads binding sites on *zo-1* gene promotor, the chromatin immunoprecipitation (ChIP)-PCR with anti-Smad2/3 antibody and multiple primer pairs covering the whole *zo-1* promoter region were implemented. Unfortunately, we failed to identify any potential binding region of Smad2/3 on the *zo-1* promoter region (Figure S1B), and we therefore presumed that TGFβ1-mediated regulation of ZO-1 was indirect, and that there might exist some other direct adaptors involved. 

**Figure 4:**
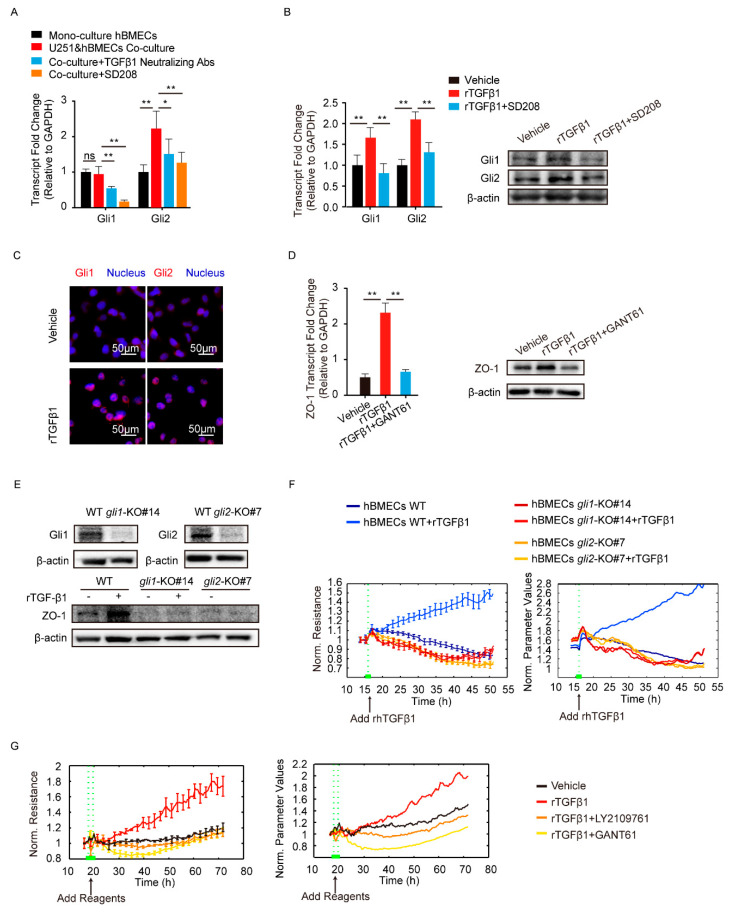
TGFβ1 enhanced ZO-1 expression via non-canonical hedgehog signaling. (**A**) The transcription of Gli1 and Gli2 in mono-culture hBMECs and the co-culture of hBMECs with or without TGFβ1 neutralizing antibody (10 μg/mL) or SD208 (10 μM). * *p* < 0.05 and ** *p* < 0.01. (**B**) The expression of Gli1 and Gli2 in response to rTGFβ1 (50 ng/mL) with or without SD208 (10 μM) pretreatment via Western blot and qPCR. ** *p* < 0.01. (**C**) Nucleus translocation of Gli1 and Gli2 in hBMECs in response to TGFβ1 (50 ng/mL) via IF. Scale indicates 50 μm. (**D**) The expression of ZO-1 in hBMECs in response to TGFβ1 (50 ng/mL) with or without GANT61 (10 μM) via Western blot and qPCR. ** *p* < 0.01. (**E**) The gli1 and gli2 deletion in hBMECs through CRISPR/Cas9, and the expression of ZO-1 in both WT cells and KO cells in response to TGFβ1. (**F**) ECIS assays analyzing the barrier resistance of the WT cells and the *gli1* or *gli2* KO cells with the addition of TGFβ1 (50 ng/mL). (**G**) ECIS assays showing the effects of TGFβ1 (50 ng/mL), together with LY2109761 (10 μM) or GANT61 (10 μM), on the barrier function of monolayer hBMECs.

TGFβ1 signaling cross-talking with hedgehog signaling has been previously reported in tumorigenesis and tumor metastasis [27]. Here, we attempted to detect the major transcription factors Gli1 and Gli2 in hedgehog signaling. The results showed a significant upregulation of Gli2 in hBMECs co-cultured with U251, compared with its mono-cultured cells, and this Gli2 upregulation was significantly blocked by the treatment of SD208 and TGFβ1 neutralizing antibody (Figure 4A). In mono-cultured hBMECs, rTGFβ1 treatment largely promoted the Gli1 and Gli2 expression and their translocation into the nucleus (Figure 4B,C), and this rTGFβ1-enhanced Gli1 and Gli2 expression was significantly suppressed by SD208 (Figure 4B). Consistently, Western blot demonstrated that SD208 injection decreased both Gli1 and Gli2 expression in mouse brains (Figure 2G and Figure S2C). Additionally, IF assay showed that the Gli2 expression in BMECs (circled with white dotted line) was decreased by SD208, while the Gli1 in BMECs (circled with white dotted line) was expressed without obviously fluctuation (Figure 3). These in vitro and in vivo data together implied that the rTGFβ1 triggered non-canonical hedgehog signaling in endothelial cells. We next employed the specific hedgehog signaling inhibitor GANT61 and found that rTGFβ1-induced upregulation of ZO-1 was suppressed (Figure 4D). Moreover, the *gli1* or *gli2* gene in hBMECs was successfully knocked out by CRISPR/Cas9 approach (Figure 4E). Compared with the rTGFβ1-induced high ZO-1 expression and high barrier resistance in wild-type cells, rTGFβ1 failed to increase ZO-1 expression and the hBMECs barrier resistance in both *gli1*-KO and *gli2*-KO cells (Figure 4E,F). Meanwhile, ECIS data indicated that both the TGFBRI/II inhibitor LY2109761 and the hedgehog signaling inhibitor GANT61 could completely block the rTGFβ1-enhanced barrier function (Figure 4G). These observations therefore evidenced that rTGFbeta1-mediated barrier function enhancement required the involvement of Gli1 and Gli2.

### 3.4. TGFβ1 Triggered the SMADS/Gli2/ZO-1 Regulatory Cascades

**Figure 5:**
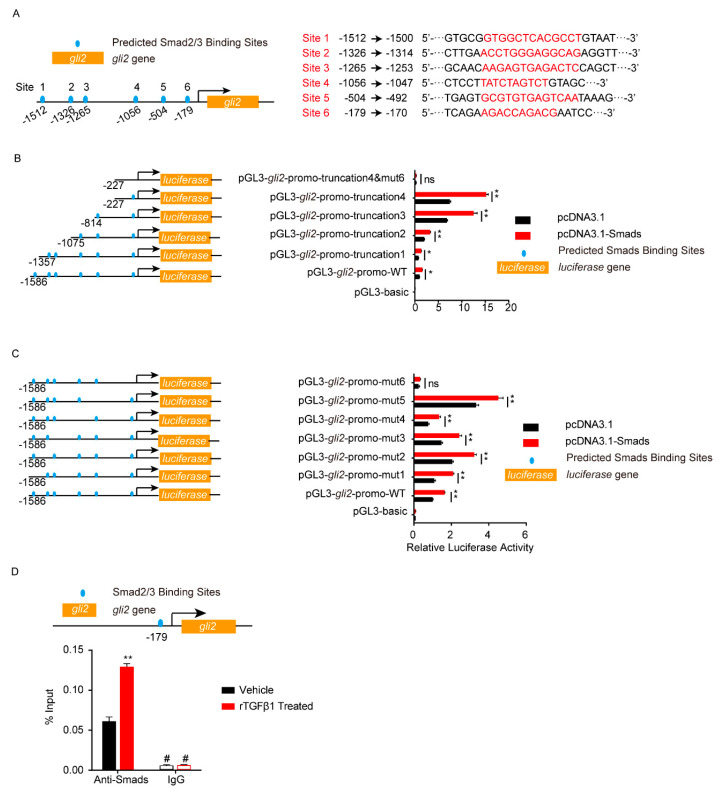
Analysis of Smad2/3 binding sites on *gli2* promotor by dual-luciferase reporter assays. (**A**) Schematic of the 6 predicted Smad2/3 binding sites on *gli2* promotor (left) and their binding sequences accordingly (right). The binding sites were located at −1512 to −1500 (site 1), −1326 to −1314 (site 2), −1265 to −1253 (site 3), −1056 to −1047 (site 4), −504 to −492 (site 5), and −179 to −170 (site 6) of the gli2 promotor region. (**B**–**C**) The *gli2* luciferase activity was tested by applying a series of truncations (**B**) and site-targeted mutations (**C**) to the *gli2* promoter, along with pcDNA3.1-Smad2 and pcDNA3.1-Smad3 and pRL-TK plasmids. The specific constructs used in the truncation assays (**B**) included the pGL3-basic vector, pGL3-*gli2*-promo-WT (containing promotor region from −1568 to +35), pGL3-*gli2*-promo-truncation1 (from −1357 to +35), pGL3-*gli2*-promo-truncation2 (from −1075 to +35), pGL3-*gli2*-promo-truncation3 (from −814 to +35), pGL3-*gli2*-promo-truncation4 (from −227 to +35), and pGL3-*gli2*-promo-truncation4&mut6 (from −227 to +35 which lacks site 6). The specific constructs used in the site-mutation assays (**C**) included the pGL3-basic vector, pGL3-*gli2*-promo-WT (containing all 6 sites), pGL3-*gli2*-promo-mut1 (lack of site 1), pGL3-gli2-promo-mut2 (lack of site 2), pGL3-*gli2*-promo-mut3 (lack of site 3), pGL3-*gli2*-promo-mut4 (lack of site 4), pGL3-*gli2*-promo mut5 (lack of site 5), and pGL3-*gli2*-promo-mut6 (lack of site 6). The luciferase activities were determined, and are presented as the ratios of firefly luciferase activity and renilla luciferase activity. The assays were performed with 3 replicates, and data are presented as mean ± SEM. * *p* < 0.05, ** *p* < 0.01. ns, no significance. (**D**) ChIP-qPCR validation of the Smad2/3 binding to the gli2 promotor around −179 in hBMECs treated by TGFβ1 (50 ng/mL). ChIP was performed with anti-Smad2/3 antibody and rabbit IgG was employed as the negative control. # indicates lower than the detection limits. ** *p* < 0.01.

**Figure 6:**
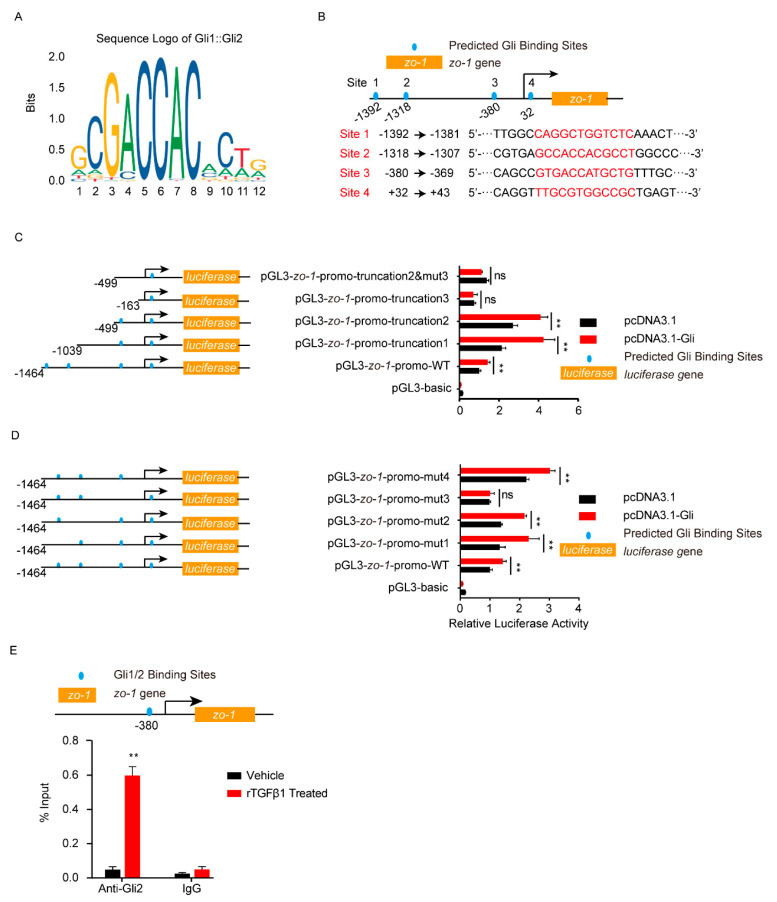
Analysis of Gli1/2 binding sites on *zo-1* promotor by dual-luciferase reporter assays. (**A**) The sequence logo of the Gli1/2 binding motif. (**B**) Schematic of the 4 predicted Gli1/2 binding sites on *zo-1* promotor and their binding sequences accordingly. The binding sites were located at −1392 to −1381 (site 1), −1318 to −1307 (site 2), −380 to −369 (site 3), and +32 to +43 (site 4) of the *zo-1* promotor. (**C**,**D**) The zo-1 luciferase activities tested by applying a series of truncations (**C**) and site-targeted mutations (**D**) to the *zo-1* promoter, along with pcDNA3.1-Gli1, pcDNA3.1-Gli2, and pRL-TK plasmids. The specific constructs used in the truncation assays (**C**) included pGL3-basic vector, pGL3-*zo-1*-promo-WT (containing promotor region from −1464 to +167), pGL3-*zo-1*-promo-truncation1 (from −1039 to +167), pGL3-*zo-1*-promo-truncation2 (from −499 to +167), pGL3-*zo-1*-promo-truncation3 (from −163 to +167), and pGL3-*zo-1*-promo-truncation2&mut3 (from −499 to +167 which lacking site 3). The specific constructs used in the site-mutation assays (**D**) included pGL3-basic vector, pGL3-*zo-1*-promo-WT (containing all 4 sites), pGL3-*zo-1*-promo-mut1 (lack of site 1), pGL3-*zo-1*-promo-mut2 (lack of site 2), pGL3-*zo-1*-promo-mut3 (lack of site 3), and pGL3-*zo-1*-promo-mut4 (lack of site 4). The luciferase activities were determined, and are presented as the ratios of firefly luciferase activity and renilla luciferase activity. The assays were performed with 3 replicates, and data are presented as mean ± SEM. ** *p* < 0.01. ns, no significance. (**E**) ChIP-qPCR validation of the Gli1/2 binding to the zo-1 promotor around −380 in hBMECs treated by TGFβ1 (50 ng/mL). ChIP was performed with anti-Gli2 antibody, and rabbit IgG was used as the negative control. ** *p* < 0.01. The assays were performed with at least 3 replicates, and data were presented as mean ± SEM.

Smad2/3 or Gli2 functioned as transcription factors, so next we analyzed the potential binding sites on their target genes. As shown in Figure 5A, six potential Smad2/3 binding sites were predicted in the *gli2* promotor region (site 1–6). The gli2 promotor region, including the full-length promotor region and a series of truncation and site-mutations, were cloned and constructed. Dual-luciferase reporter assays from both truncation and site-mutation all indicated that the site 6, 5′-AGACCAGACG-3′ (from −179 to −170), was the Smad2/3 binding region on *gli2* promotor (Figure 5B,C). With ChIP-qPCR, it was obvious that the *gli2* promotor range from −179 to −170 (5′-AGACCAGACG-3′) bound by Smad2/3 was significantly increased in response to rTGFβ1 (Figure 5D), which further evidenced the regulation of *gli2* by Smad2/3. Likewise, the dual-luciferase reporter assays supported the site 3, 5′-GTGACCATGCTG-3′ (from −380 to −369), was the key Gli binding site on *zo-1* promotor (Figure 6A–D), and ChIP-qPCR also supported this regulation of *zo-1* by Gli1/2 (Figure 6E). 

Taken together, these findings firmly revealed that TGFβ1-Smad2/3 triggered the activation of non-canonical hedgehog signaling, which led to nucleus translocation of transcription factor Gli1/2 and its regulation by the TJ protein ZO-1, and therefore enhanced endothelial barrier function.

## 4. Discussion

The cell-to-cell communication between astrocytes and the endothelium is essential for BBB’s integrity and CNS homeostasis. As early as 1987, astrocytes were shown to support the formation, extent, and configuration of the junctional complex in endothelium by co-culturing primary bovine BMECs on a confluent bed of primary rat astrocytes [34]. In the last few decades, a few effectors participating in astrocyte–endothelium communication have been identified. For example, astrocyte-derived GDNF activated the GFRalpha1 in porcine BMECs, leading to higher TEER and lower permeability of the endothelium [10]. In the stroke model, astrocytes significantly increased the capillary tube-like formation of BMECs via Ang1/Tie2 and Flk1 expression [35], and astrocyte-derived TGFβ1 reduced tPA and TM transcription in the co-cultured BMECs, which promoted the angiogenesis and vascular stabilization after stroke [36]. Conversely, the secreta from endothelium was also reported to modulate the behavior of astrocytes. For instance, LIF, an important secretory effector of BMECs, has been shown to enhance astrocytes’ differentiation of neural precursor cells through promoting STAT signaling activation and GFAP expression [37]. Such astrocyte–endothelium communication via diverse effectors has been reported to be increasingly involved in different physiological and pathological processes, including the BBB’s functions. In this research, we similarly observed an increased TEER of hBMECs when co-cultured with U251 astrocytes, further supporting the contribution of cell-to-cell communication to BBB function maintenance.

Based on our previous transcriptomic and secretory proteomic data, and the in vitro ECIS assay and Western blot, we hypothesized the astrocyte-derived TGFβ1 might be a key effector that mediates this astrocyte–endothelium communication. The addition of TGFβ1 significantly increased the ZO-1 expression and elevated the barrier resistance in a dose-dependent manner, and blocking this TGFβ1 signaling largely attenuated ZO-1 expression. As mentioned above, astrocyte-derived Ang1/2 and GDNF were also reported as effectors to maintain the low permeability of BBB [10,12]. However, the enhancement of hBMECs by Ang1/2 or GDNF was not additionally observed in the current co-culture model when TGFβ1 was blocked by SD208 or when we used a neutralizing antibody. As for Ang1/2, they were reported to maintain the barrier function through upregulating the occludin expression in BMECs [12]. Additionally, in our study, TGFβ1 was found to modulate ZO-1 expression in BMECs. Both occludin and ZO-1 are recognized as components of tight junctions in BBB, and disruption of either would weaken the BBB, which might be the reason why we did not notice the Ang1/2 effects in the co-culture model. As for GDNF, it was reported to modulate the barrier’s function through triggering cytoskeleton rearrangement in BMECs [38]. At this time, we could not tell the reason for the GDNF effects not being observed in our co-culture model when TGFβ1 was blocked. There might be potential interplay between TGFβ1 signaling and GDNF signaling. For instance, it was reported that GDNF expression in hGL cells relied on TGFβ1 [39]. Anyway, the results in this work supported the concept that astrocyte-derived TGFβ1 helped to enhance the BBB’s function. Aside from the BBB, TGFβ1 has also been shown to have other protective effects on the CNS. For instance, TGFβ1 was reported to regulate the proliferation and activation of macrophages, microglia, and T cells, which participate in oligodendrocytes’ myelination [40], and treatment with anti-TGFβ1 antibody would exacerbate the symptoms in experimental allergic encephalitis [41,42]. A study on *tgfbr2*-deficient mice revealed that the silencing of TGFβ1 signaling in mice microglia led to glial activation and neuronal loss [43]. Besides, TGFβ1 was reported to protect neurons from apoptosis. On the one hand, TGFβ1 in neurons activated the Erk pathway to phosphorylate the pro-apoptosis protein Bad, and thus acted as a neuroprotective effector [44]. On the other hand, TGFβ1 in astrocytes directly induced PAI-1 transcription [45], which is an inhibitor of apoptosis induced by t-PA. Actually, TGFβ1 was observed to protect neurons in vitro from apoptosis induced by NMDA, AMPA, and kainite with a manner dependent on PAI-1 in the presence of astrocytes [46]. Our data in this work, together with the previous study above, suggest TGFβ1 as a potential target in CNS disease therapy. 

With further investigation, we observed cross-talking between the TGFβ1 pathway and hedgehog signaling, and demonstrated the Gli1/2 promotion of ZO-1 expression induced by TGFβ1 in hBMECs. As mentioned previously, the roles of hedgehog signaling and its key transcription factors Gli1/2 have been previously reported in multiple physiological and pathological processes. For example, hedgehog signaling was shown to be essential for the normal development of adrenal glands, and the lack of Shh (one of the hedgehog signaling ligands) may cause adrenal hypoplasia and thin capsule [47]. This signaling also acts importantly in the angiogenesis process, in which the transcription factor Gli1 in artery endothelial cells promotes the expression of Ang1, Ang2, and VEGF, and thus triggers vascular remodeling by activating Notch signaling [48]. However, the angiogenic activity did not seem to be involved in the ZO-1 modulating process in BMECs. The angiogenesis effectors Ang1, Ang2, and VEGF in hBMECs did not show significantly differential transcription under mono-culture and co-culture conditions (Figure S1C). Besides, the cascade is also involved in certain infectious diseases, such as malaria. The plasmodium could activate hedgehog signaling in hepatic stellate cells to induce hepatic fibrogenesis and severe hepatic inflammation [49]. In this study, we demonstrated that TGFβ1 activated the hedgehog signaling in endothelial cells through Smad2/3 regulation of Gli1/2, which enhances the TJs and promotes the endothelial barrier function. This finding suggests that astrocytes play important roles in maintaining the endothelial barrier function through TGFβ1-mediated astrocyte–endothelium communication.

## 5. Conclusions

**Figure 7:**
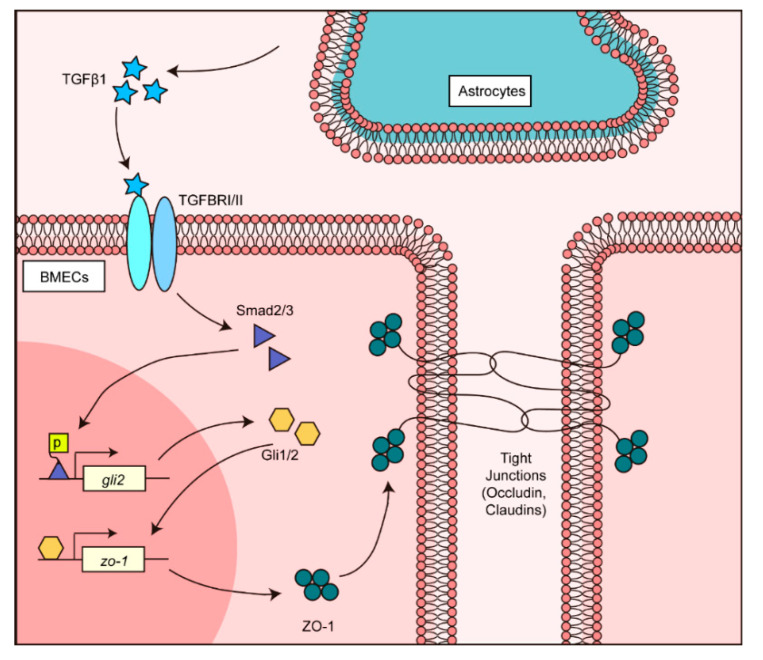
Schematic representation of the TGFβ1-mediated intercellular communication between astrocyte and BMECs in BBB. Astrocyte-derived TGFβ1 triggered the TGFβ1-TGFBRII-Smad2/3-Gli1/2-ZO-1 axis in BMECs and maintained the normal BBB function.

In summary, we demonstrated that TGFβ1 mediates cell-to-cell communication that contributes to the BBB’s normal maintenance. As presented in Figure 7, the astrocyte-derived TGFβ1 activated transcriptional factors Smad2/3 in endothelial cells and triggered non-canonical hedgehog signaling activation. Activated hedgehog transcriptional factor Gli1/2 next modulated the *zo-1* promotor, and enhanced the TJ protein ZO-1 expression in BMECs. This work described novel cell-to-cell communication in the BBB involving TGFβ1, and extended our knowledge about the BBB’s functional maintenance. This finding may also suggest the possibility of using TGFβ1 and hedgehog signaling cascades to assist in the treatment of BBB dysfunction in future.

## Data Availability

The data presented in this study are available on request from the corresponding author.

## Supplementary Materials

The following are available online at https://www.mdpi.com/2076-3425/11/1/77/s1. Figure S1: TGFβ1 and TGFBRI/II expression in BMECs. Figure S2: Quantitative analyses of the Western blots. Figure S3: Detection of Smad2/3 interacting with *zo-1* promotor. Table S1: Primers used for the qPCR assays. Table S2: Primers for CDS cloning and promotor region amplification in the dual-luciferase reporter assays. Table S3: Primers used in ChIP-PCR/qPCR assays.
