# Supplementary material for: Astrocyte-Derived TGFβ1 Facilitates Blood–Brain Barrier Function via Non-Canonical Hedgehog Signaling in Brain Microvascular Endothelial Cells
Source: Brain Sci. 2021 Jan 8;11(1):77. doi: 10.3390/brainsci11010077 (PMC7826596; doi:10.3390/brainsci11010077)
Supplement: Supplementary file 1 [file brainsci-11-00077-s001.pdf]

Supplemental Figures

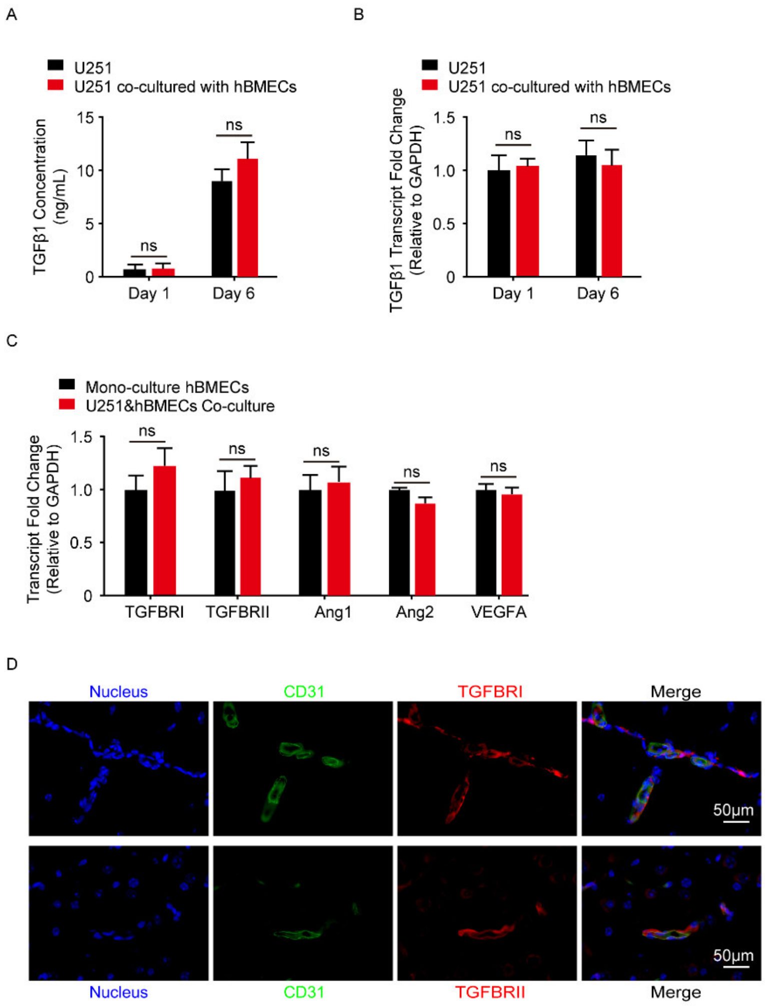

Figure S1. TGFβ1 as well as TGFBRI/II expression in BMECs. (A) ELISA detection of TGFβ1 concentration in U251 with or without hBMECs co-cultivation at Day1 and Day6. ns, no significance. (B) qPCR detecting the TGFβ1 transcription in U251 with or without hBMECs co-cultivation at Day1 and Day6. ns, no significance. (C) qPCR detected the TGFBRI, TGFBRII, Ang1, Ang2, and VEGFA transcription in hBMECs under mono-culture condition or co-culture condition. ns, no significance. (D) The expression of TGFBRI/II on BMECs of the mice via IF. BMECs were marked with CD31 in green. Scale bars indicated 50μm.

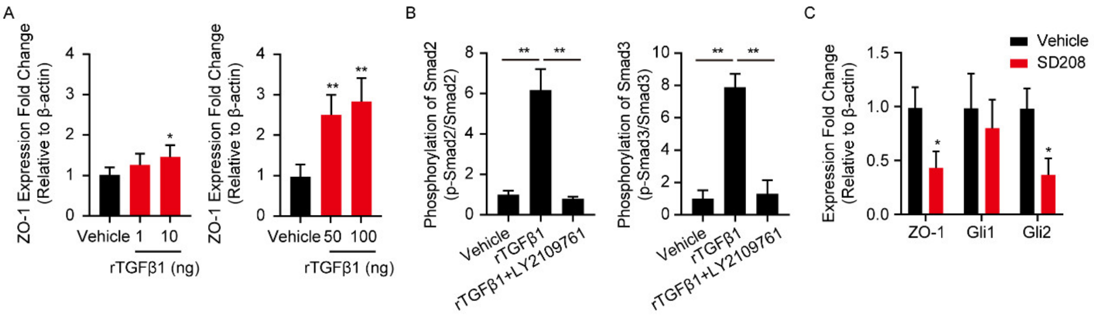

Figure S2. Quantitative analyses of the Western blots. (A) Quantitative analysis of the blots in Figure 2C. **p<0.01. (B) Quantitative analysis of the blots in Figure 2F. **p<0.01. (C) Quantitative analysis of the blots in Figure 2G. **p<0.01.

A

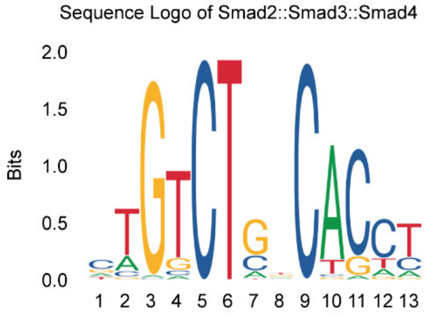

B

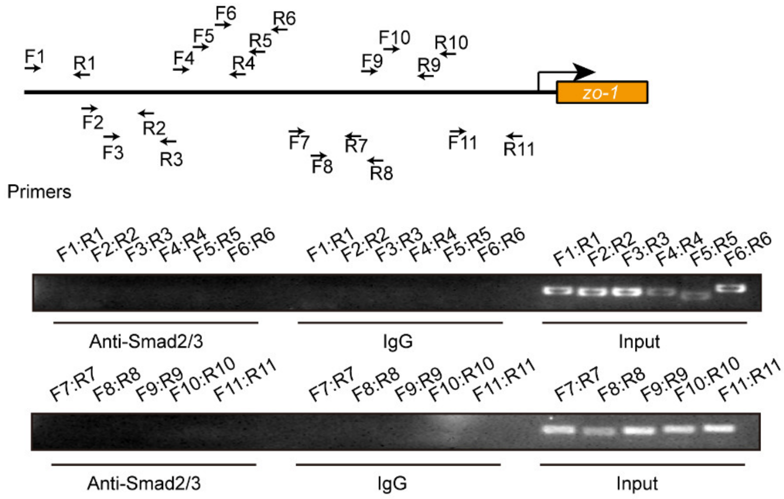

Figure S3. Detection of Smad2/3 interacting with *zo-1* promotor. (A) The sequence logo of the Smad2/3/4 binding motif. (B) Schematic showing a series of Smad2/3 primers binding as well as amplification on the *zo-1* promotor region during the ChIP-PCR process. The ChIP procedure was performed with anti-Smads antibody. Rabbit IgG was set as the negative control, and Input was the positive control without IP.

Supplemental Tables

Table S1. Primers used for the qPCR assays.

Primer name	Sequence (5′ to 3′)
zo-1-F	GACTTAAAGCTGCCTCAACAGA
zo-1-R	GGTTTGTTTCAGGCGAAAGG
ocln-F	TTAACTTCGCCTGTGGAT
ocln-R	AGTGATCTTGCTCTGTTCT
tgfbr1-F	ACGGCGTTACAGTGTTTCTG
tgfbr1-R	GCACATACAAACGGCCTATCTC
tgfbr2-F	GTAGCTCTGATGAGTGCAATGAC
tgfbr2-R	CAGATATGGCAACTCCCAGTG
ang1-F	AGCGCCGAAGTCCAGAAAAC
ang1-R	TACTCTCACGACAGTTGCCAT
ang2-F	AACTTTCGGAAGAGCATGGAC
ang2-R	CGAGTCATCGTATTCGAGCGG
vegfa-F	TGCCTTGCTGCTCTACCT
vegfa-R	GACATCCATGAACTTCACCACTT
gli1-F	GCTAGAGTCCAGAGGTTC
gli1-R	GTGGTGAGTAGACAGAGG
gli2-F	AGCAGCAGCAACTGTCTGAGTGA
gli2-R	GACCTTGCTGCGCTTGTGAA
gapdh-F	CAACAGCCTCAAGATCATCAG
gapdh-R	GAGTCCTTCCACGATACCA

Table S2 Primers for CDSs cloning and Promotor region amplification in the dual-luciferase reporter assays.

Primer name	Sequence (5′ to 3′)
Smad2-CDS-F	CGGGGTACCCATGTCGTCCATCTTGCCATTC
Smad2-CDS-R	CCGGAATTCTGATGAGACCTCAAGTGCTGTT
Smad3-CDS-F	CGGGGTACCAGCCATGTCGTCCATCCTG
Smad3-CDS -R	CCGGAATTCACAATGGGTTGAGTAGAGTTCC
Gli1-CDS -F	CCCAAGCTTATGTTCAACTCGATGACCCCAC
Gli1-CDS -R	GATATCTTAGGCACTAGAGTTGAGGAATTCTG
Gli2-CDS -F	CCCAAGCTTATGGAGACGTCTGCCTCAGCC
Gli2-CDS -R	CCGGAATTCCTAGGTCATCATGTTCAGGAACTTG
zo-1-promo-F	CGGGGTACCGGTACCACAGGCACGCGC
zo-1-promo-R	CCGCTCGAGCTCGAGAGCAGCACCCGTG
gli2-promo-F	CGGGGTACCCTCCAGGTGTCCAGCCAAAA
gli2-promo-R	CCCAAGCTTTCCATCTCAGCCGCTCATC
gli2-promo truncation1-F	CGGGGTACCAGCTACTCAGGATTGCAGGAG
gli2-promo truncation1-R	CCCAAGCTTTCCATCTCAGCCGCTCATC
gli2-promo truncation2-F	CGGGGTACCGCCACTTTCCATCCCTCCTTAT
gli2-promo truncation2-R	CCCAAGCTTTCCATCTCAGCCGCTCATC
gli2-promo truncation3-F	CGGGGTACCTACTCAAAGAAGCCAGCCTCCT
gli2-promo truncation3-R	CCCAAGCTTTCCATCTCAGCCGCTCATC
gli2-promo truncation4-F	CGGGGTACCCCGCCACTCATATCAGTAGGAA
gli2-promo truncation4-R	CCCAAGCTTTCCATCTCAGCCGCTCATC
zo-1-promo truncation1-F	CGGGGTACCACCATTGTCTAAAGCCTGATGT
zo-1-promo truncation1-R	CCGCTCGAGCTCGAGAGCAGCACCCGTG
zo-1-promo truncation2-F	CGGGGTACCGAACGAGAGCAACGCTTCTGAC
zo-1-promo truncation2-R	CCGCTCGAGCTCGAGAGCAGCACCCGTG
zo-1-promo truncation3-F	CGGGGTACCACGGCAGCGGAAACTGTCTT
zo-1-promo truncation3-R	CCGCTCGAGCTCGAGAGCAGCACCCGTG
gli2-promo mutation 1-R	TGGCCGGTCCACGCTTAGTTTCGTTCCCATTAGGGTCGTGAAAT
gli2-promo mutation 2-F	GCAGGAGAATCACTTGAGCCGCTAAAAATTAGGTTGCGGTGAGCC
gli2-promo mutation2-R	CGTCCTCTTAGTGAACTCGGCGATTTTTAATCCAACGCCACTCGG
gli2-promo mutation3-F	CCAGCCTGGGCAACGCCGTTCGCATTGCAGCTCAAAAACAAACA
gli2-promo mutation3-R	GGTCGGACCCGTTGCGGCAAGCGTAACGTCGAGTTTTTGTTTGT
gli2-promo mutation4-F	ACTTTCCATCCCTCCTCGAATCGCGCGTAGCCTGGTCTGTTGG
gli2-promo mutation4-R	TGAAAGGTAGGGAGGAGCTTAGCGCGCATCGGACCAGACAACC
gli2-promo mutation5-F	AAAATATGTTGACTGAGTATAGCTTGCCTGCTAAAGGATGAACATTTAT
gli2-promo mutation5-R	TTTTATACAACTGACTCATATCGAACGGACGATTTCCTACTTGTAAATA
gli2-promo mutation6-F	GTGAGGTCGTTCAGAGCATGCGTCAAATCCCAGGACTAATAA
gli2-promo mutation6-R	CACTCCAGCAAGTCTCGTACGCAGTTTAGGGTCCTGATTATT
zo-1-promo mutation1-F	GTTTCACCATATTGGCCGATGTAGCGGTAAACTCCTGACCTCG
zo-1-promo mutation1-R	CGAGGTCAGGAGTTTACCGCTACATCGGCCAATATGGTGAAAC
zo-1-promo mutation2-F	GGATTACAGGCGTGATTCCTGCATACTTGGCCCACAATTCTTA
zo-1-promo mutation2-R	TAAGAATTGTGGGCCAAGTATGCAGGAATCACGCCTGTAATCC

zo-1-promo mutation3-F	CGCGGTGACAGCCCGATGTAGCGGTTTTGCCCAGTGAAGG
zo-1-promo mutation3-R	CCTTCACTGGGCAAAACCGCTACATCGGGCTGTCACCGCG
zo-1-promo mutation4-F	CGGGCCGGCAGGTTTCCTGCATACTTGAGTTGCCGGCGC
zo-1-promo mutation4-R	GCGCCGGCAACTCAAGTATGCAGGAAACCTGCCGGCCCG

Table S3. Primers used in ChIP-PCR/qPCR assays.

Primer name	Sequence (5′ to 3′)
ChIP-Smad×*gli2*-F	GCCACTCATATCAGTAGGA
ChIP-Smad×*gli2*-R	GGATTCGTCTGGTCTTCT
ChIP-Gli×*zo-1*-F1	CAAGATGTGTGGCACTTC
ChIP-Gli×*zo-1*-R1	CAGGCTTTAGACAATGGTTT
ChIP-Gli×*zo-1*-F2	GTGGCACTATACCAGATACT
ChIP-Gli×*zo-1*-R2	CCTAGACTCACTAATCTACACT
ChIP-Gli×*zo-1*-F3	GATGTTACTAAGGATTTCTGGC
ChIP-Gli×*zo-1*-R3	GGCTTTGCTAAACACCAA
ChIP-Gli×*zo-1*-F4	TTTAGCAAAGCCGTCAAC
ChIP-Gli×*zo-1*-R4	TGTCCCTCCAACTCAAAG
ChIP-Gli×*zo-1*-F5	CTTTGAGTTGGAGGGACA
ChIP-Gli×*zo-1*-R5	GCCATCAAGATTGCTGAA
ChIP-Gli×*zo-1*-F6	CGTTCGGTCAACAAGAATT
ChIP-Gli×*zo-1*-R6	CCTGAGAAACACCCTAGAG
ChIP-Gli×*zo-1*-F7	TCTTGAGGTCTAATGTGGG
ChIP-Gli×*zo-1*-R7	AAGGCTGAAACTGGTGAT
ChIP-Gli×*zo-1*-F8	ACCTCATCACCAGTTTCA
ChIP-Gli×*zo-1*-R8	CGTCATGGCTTTCATCTC
ChIP-Gli×*zo-1*-F9	GAAACAACTGTCGGGTATC
ChIP-Gli×*zo-1*-R9	GAGACTTGTCCACTTGCT
ChIP-Gli×*zo-1*-F10	AGATGAAAGCCATGACGC
ChIP-Gli×*zo-1*-R10	CGATACCCGACAGTTGTT
ChIP-Gli×*zo-1*-F11	GGACAAGTCTCTTAAGGAAAG
ChIP-Gli×*zo-1*-R11	CGGGTAACCCAAGTAACT
